# Exhumation history of the Sanshandao Au deposit, Jiaodong: constraints from structural analysis and (U-Th)/He thermochronology

**DOI:** 10.1038/s41598-017-08103-w

**Published:** 2017-08-10

**Authors:** Xuan Liu, Hong-Rui Fan, Noreen J. Evans, Kui-Feng Yang, Martin Danišík, Brent I. A. McInnes, Ke-Zhang Qin, Xue-Feng Yu

**Affiliations:** 1 Key Laboratory of Mineral Resources, Institute of Geology and Geophysics, Chinese Academy of Sciences, Beijing, 100029 China; 2 John de Laeter Centre, Department of Applied Geology/Applied Physics, Curtin University, Perth, WA 6845 Australia; 3 College of Earth Science, University of Chinese Academy of Sciences, Beijing, 100049 China; 4 Shandong Geological Sciences Institute, Jinan, 250013 China

## Abstract

The Sanshandao gold deposit contains an estimated Au resource of >1500 tons, however little is known about the history of exhumation, and the magnitude of displacement on the ore-hosting fault. Structural measurement revealed two phases of normal and one phase of sinistral movement on the fault. Despite of intra-sample dispersions, (U-Th)/He ages from two sub-vertical profiles show decreasing trends from the surface down to −3560 m (zircon: 123 Ma to 55 Ma; apatite 103 Ma to 0.3 Ma). Over-dispersion of AHe ages likely reflects the presence of undetected inclusions. According to the age-depth pattern, we infer that the deposit underwent an early phase of rapid cooling in the late Early Cretaceous, which was followed by a short period of thermal stagnation and a revived rapid cooling between 75 Ma and 55 Ma in response to a combined effects of late normal movement and erosion. Since the Eocene, the deposit has experienced a slow monotonic cooling. Exhumation magnitude estimates suggest that the deposit have been denudated > 5.1 km. The two phases of normal displacement along the fault occurred in the late Early Cretaceous and Late Cretaceous to Paleocene, leading to a total offset magnitude of 0.5–2.3 km.

## Introduction

Since first being recognized as a potential thermochronometer by Zeitler *et al*.^1^, (U-Th)/He dating has been applied in a wide range of studies, such as landscape evolution^2^, hydrocarbon exploration^3^ and ore preservation investigation^4^. Understanding the timing of exhumation and exposure of hypogenic Au deposits is of particular interest to the exploration community given the implications for preservation of ore deposits and the host terrain^5^. Zircon and apatite (U-Th)/He thermochronometers (ZHe and AHe, respectively) are sensitive to temperature ranges of 210 °C to 130 °C^6^ and 80 °C to 40 °C^7^, respectively, spanning the low temperature history of a hydrothermal Au deposit, and providing important insights on these processes.

The Sanshandao Au deposit is a typical fault-controlled one, and two interesting features make it attractive for thermochronology studies: (i) previous fluid inclusion thermobarometric results suggested pervasive denudation (>4 km)^8^, but the exact timing and mechanism of the exhumation has not been previously studied; and (ii) hydrothermal alteration envelops both the hanging wall and footwall, but orebodies have only been discovered in the footwall. Determining the fault offset direction and quantifying the offset magnitude will assist exploration and impact reserve estimation. The present study reports novel results from zircon and apatite (U-Th)/He thermochronology and structural analysis, and proposes a thermal model for deposit evolution and exhumation. The structural and tectonic processes responsible for exhumation and the implications for deposit-scale and regional gold exploration are discussed.

## The Jiaodong gold province and Sanshandao deposit

**Figure 1:**
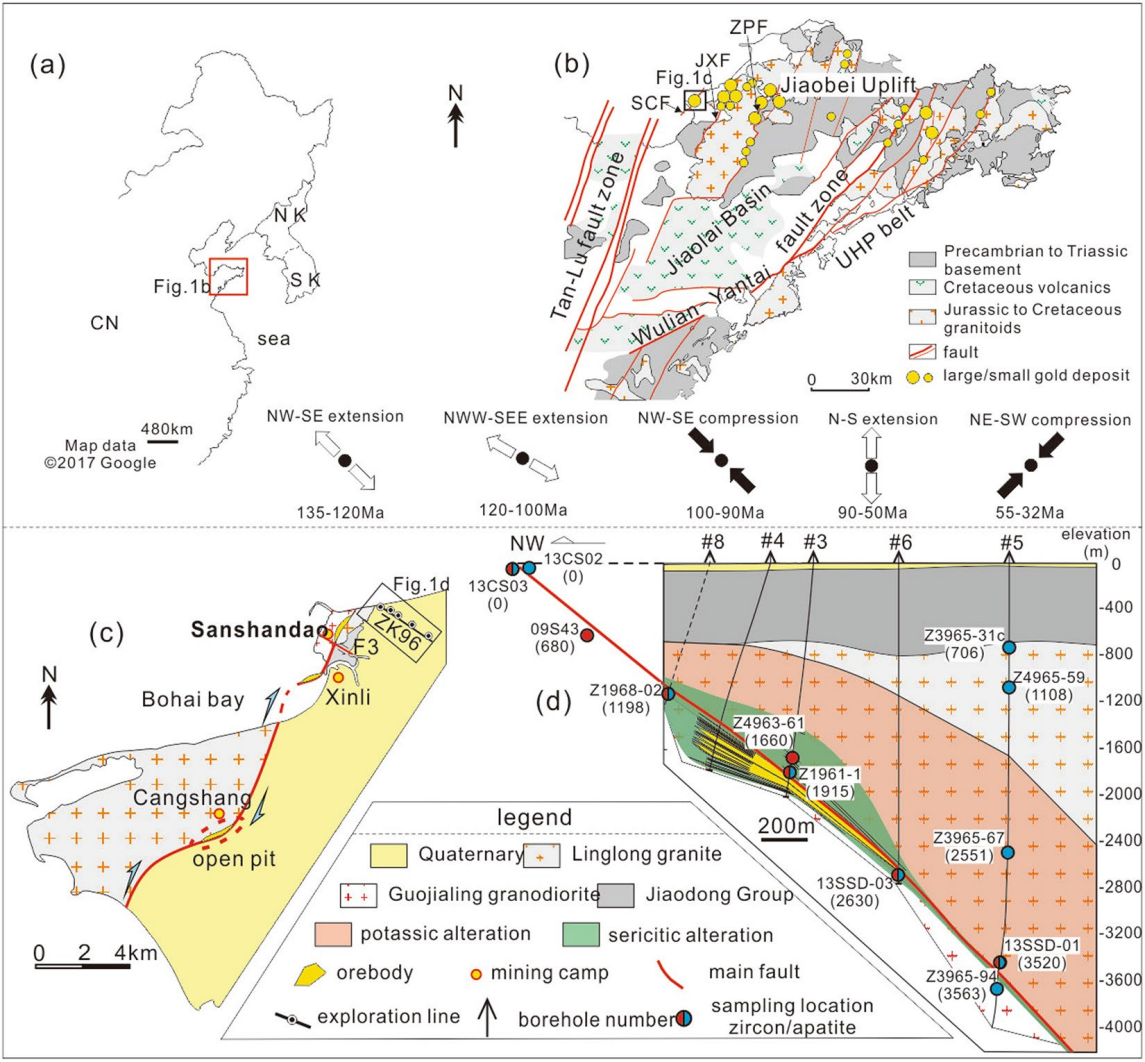
Maps showing the location (**a**) and general geology (**b**) of Jiaodong; Geologic maps showing the Sanshandao-Cangshang belt (**c**) and sampling localities (**d**); CN: China, NK: North Korea, SK: South Korea, SCF: Sanshandao-Cangshang fault, JXF: Jiaojia-Xincheng fault, ZPF: Zhaoyuan-Pingdu fault, UHP: ultra-high pressure; the paleostress directions in Fig. 1b are from ref. 20. Note samples 13CS02 and 13CS03 were collected from the Cangshang open pit, and 09S43 was collected from underground tunnel at Sanshandao. Numbers in the parentheses after the sample number represent sampling depth. Figure 1a was made with data from Google Maps (https://www.google.com/maps), and was reproduced using CorelDRAW × 6 (http://www.coreldraw.com/en/). Figure 1b,c,d were adapted from ref. 27.

The Jiaodong peninsula (Fig. 1a) is the largest gold producing province in China (Au reserves of >3000 tons)^9^. It is bordered by the Tan-Lu fault zone to the west and comprises the Sulu ultra-high pressure (UHP) orogenic belt and Jiaobei terrain which are separated by the Wulian-Yantai Fault (Fig. 1b). The Sulu belt consists of Neoproterozoic metamorphic basement, Triassic UHP rocks and Mesozoic granitoids^10^. The Jiaobei terrain is subdivided into the northern Jiaobei Uplift and southern Jiaolai Basin. The Jiaobei Uplift consists of Precambrian metamorphic basement and widespread Mesozoic granitoids while the Jiaolai Basin is filled with a basal lacustrine sediment sequence (Laiyang Group) (130 Ma), central volcanic rocks (Qingshan Group) (120–105 Ma) and an upper lacustrine sediment sequence (Wangshi Group) (~80 Ma)^11^. A large number of subordinate NE- and NNE-trending faults were developed and these host over 50 gold deposits (Fig. 1b). Gold mineralization occurs in the second-order faults as disseminated ores or in the third-order extensional cracks as auriferous quartz veins^12^. Extensive geochronological studies have revealed that the deposits were formed at 120 ± 10 Ma^13^. In terms of the tectonic evolution, Zhang *et al*.^14^ proposed that Jiaodong experienced two episodes of extension (NW-SE extension at 135–120 Ma and NWW-SEE extension at 120–100 Ma) and a short period of NW-SE compression from 100–90 Ma, followed by N-S extension in the Late Cretaceous to Early Paleocene and subsequent NE-SW compression at the end of the Paleocene (Fig. 1b). Consistent with this regional interpretation, Deng *et al*.^15^ suggested that the NE-trending Jiaojia-Xincheng fault (JXF) experienced normal faulting from 135 Ma to 120 Ma, and a sinistral slip from 120–110 Ma, which was followed by normal displacement at ca. 110 Ma. From 80–60 Ma, the fault acted as a normal fault, followed by a dextral reactivation at ca. 55 Ma.

The Sanshandao Au deposit is hosted by the Sanshandao-Cangshang fault (SCF), and to its south are two mining camps (Xinli and Cangshang) (Fig. 1c). To its north, the fault extends into Bohai Bay, and recent exploration drilling discovered large orebodies with 470 tons Au resource in the undersea region^16^. Overall, the belt hosts more than 1500 tons of Au. The ore bodies are commonly several meters thick and localized only in the footwall rocks. They comprise disseminated-style sulfides enclosed by envelopes of strong silicification proximal to the fault, trending to sericitization and potassic alteration with increasing distance from the fault^17^. The Linglong biotite granite is the major host to shallow orebodies, while at depths below 1‒1.5 km, orebodies are mainly hosted in the Guojialing granodiorite. Laser ablation ICPMS zircon U-Pb dating suggested that the Linglong granite and Guojialing granodiorite were emplaced at ca. 160 Ma and 130 Ma, respectively^18^. Thermobarometric results indicate that Guojialing formed at ca. 13 km depth^19^. Ar-Ar and Rb-Sr dating on hydrothermal sericite yielded mineralization ages of 121–117 Ma^20, 21^. Temperature measurements in the drill hole ZK96–5 indicated a modern geothermal gradient of about 25 °C/km.

## Sampling Technique

Samples were collected at 500 m intervals, yielding sample pairs at similar depths in both the hanging wall and footwall. In total, twelve samples were collected from underground tunnels, drill holes and from the Cangshang open pit, covering a depth range of 0 to −3563 m (Fig. 1d). Ten samples were selected for AHe dating (five from the footwall and five from the hanging wall) and seven samples were selected for ZHe dating (four from footwall and three from hanging wall). Four samples of altered Guojialing granodiorite were taken from the footwall and eight samples of altered Linglong biotite granite were taken from the hanging wall and footwall. A detailed description of sampling locality, depth and lithology is provided in Table 1.

**Table 1:** A summary of sampling locality, depth and lithology.

Sample N.O.	Locality	Profile	Depth (m)	Relative location	Lithology
13CS02	Cangshang open pit	along the fault	0	hanging	altered biotite granite
13CS03			0	foot	
09S43	Sanshandao underground tunnel		−680	foot	
Z1968-02	ZK96 exploration line		−1198	foot	altered granodiorite
Z4963-61			−1660	hanging	altered biotite granite
Z1961-01			−1914.7	foot	altered granodiorite
13SSD-03			−2630	foot	
Z3965-31c		drilling hole (ZK96-5)	−706.3	hanging	altered biotite granite
Z4965-59			−1107.6	hanging	
Z3965-67			−2551.1	hanging	
13SSD-01		intersection	−3520.4	hanging	
Z3965-94			−3563.4	foot	altered granodiorite

## Results

### Structural analysis

**Figure 2:**
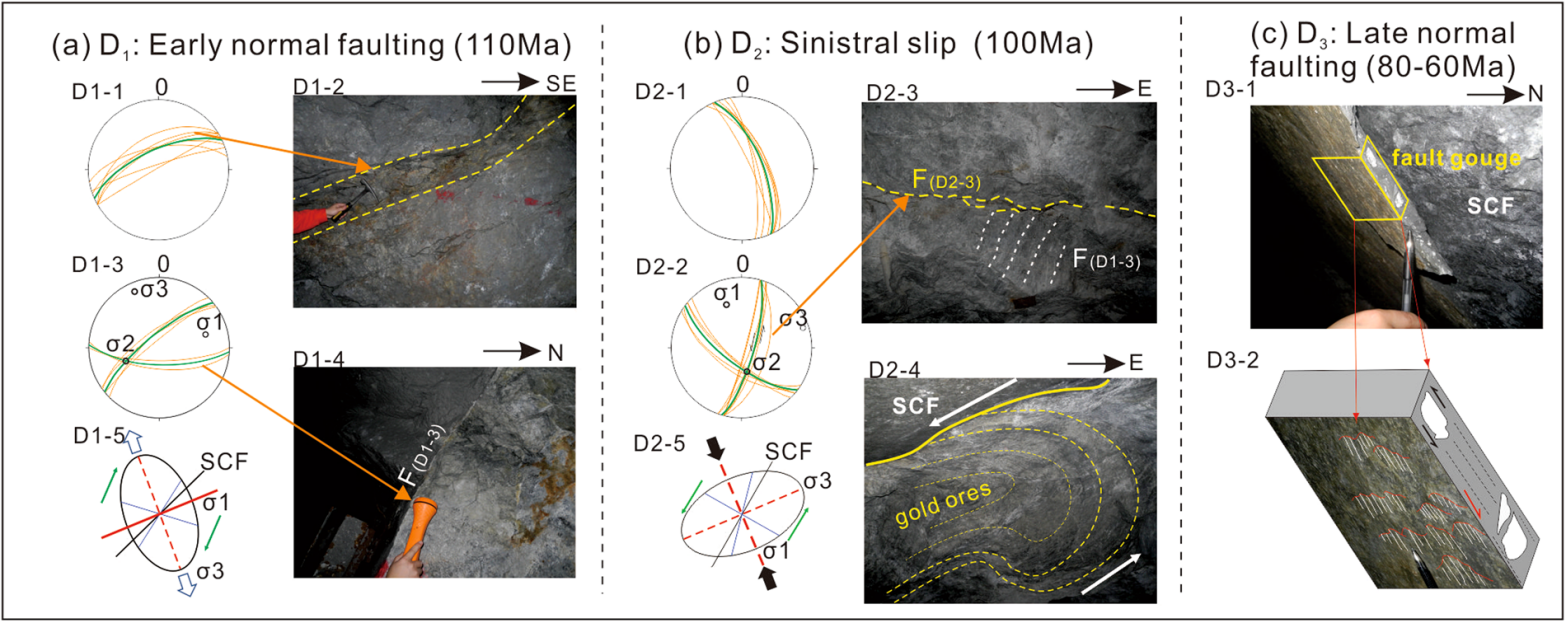
(**a**) D1-1 is a Stereographic Nets representative of the measurements of the extensional fissures as shown in D1-2; D1-3 is a Stereographic Nets representative of the measurements of conjugated joints shown in D1-4; D1-5 is the paleostress analysis ellipsoid for D1-1 and D1-2; (**b**) D2-1 is a Stereographic Nets representative of the measurements of the sinistral slip faults; D2-2 is a Stereographic Nets representative of the measurements of conjugated shear joints as shown in D2-3. Note the shear joint truncated early joints from D_1_; D2-4 is a photo of the folding of ore body; D2-5 is a paleostress analysis ellipsoid for D2-1 and D2-2; (**c**) D3-1 is a photo of the main fault and its gouge; D3-2 is a sketch of steps on the fault plane.

The post-mineralization (<120 Ma) kinetic history of the SCF fault was reconstructed using detailed structural measurements (Table 2). Two phases of normal faulting and an intervening phase of sinistral movement were recognized. During the early phase of normal faulting (D_1_), a series of subordinate NE-trending, NW-dipping extensional faults were formed, which were commonly 10 to 30 centimeters wide and consisted of randomly-distributed breccias (Fig. 2a D1-1, 2). These extensional faults are coupled to a set of conjugated joints, which dip to south and northwest, respectively, at high angles (Fig. 2a D1-3, 4). These structures indicate a NNW-SSE direction for the extensional stress, which is consistent with the regional stress suggested by Zhang *et al*.^14^ and the kinetic state of the JXF at 110 Ma^15^ (Fig. 2a D1-5). In addition, the extensional faults are filled with hydrothermal calcite that formed during the late stages of gold mineralization. Following D_1_, a NW-SE trending principal compressive stress led to sinistral displacement (D_2_), NW-trending extensional fault that was named “F3” (Fig. 1; Fig. 2b D2-1), and conjugated joints (dipping to southeast and southwest, respectively) (Fig. 2b D2-2) and the folding of ore bodies (Fig. 2b D2-4), which is consistent with regional stress at the beginning of 100 Ma as suggested by Zhang *et al*.^14^. The NE-trending conjugate joints of D_2_ (e.g. F(D2-3) in Fig. 2b D2-3) truncated the E-W trending joints of D_1_ (e.g. F(D1-3) in Fig. 2a D1-3 and Fig. 2b D2-5), suggesting that the sinistral movement occurred later. The sinistral shear fault F3 crosscuts orebodies, further supporting the above interpretation (Fig. 1). The folding of ore bodies indicates that the deformation occurred after gold mineralization and after the D_1_ deformation event. Stress analysis indicates a NW-SE compression, which resembles the regional stress at around 100 Ma (Fig. 2b D2-5). A late stage of normal displacement (D_3_) on the SCF was identified through observations of striations and steps on the SCF fault plane (Fig. 2c D3-1, 2). The orientation of the rotating porphyroclasts within the gouge records the D_2_ thrusting movement (Fig. 2c D3-1), supporting the interpretation that D_3_ post-dated D_2_. Deng *et al*.^15^ reported illite K-Ar ages of 83 Ma to 68 Ma for fault gauge for the JXF, and interpreted them as the timing of the second normal movement along the fault. Since the SCF and JXF are cogenetic, it is likely that the D_3_ normal faulting occurred in this period. In addition, the Jiaodong region experiences an N-S extension between 90 Ma to 50 Ma^14^. Under this stress field, the SCF would have experience normal movement, as observed. Therefore, we suggest that the late normal movement along the SCF likely occurred at ca. 80–60 Ma.

**Table 2:** A summary of the structural measurements in the Sanshandao - Cangshang region.

Locality	Level/Longitude	Exploration Line/Latitude	Generation	Dip	Dip angle
Sanshandao surface	37°24′33.9″	119°56′50.6″	Fig. 2a D1-1	330°	88°
	37°24′34.4″	119°56′50.9″		330°	67°
	37°24′32.3″	119°56′46.0″		342°	84°
	37°24′30.4″	119°56′49.6″		350°	64°
	37°24′22.2″	119°56′24.6″		340°	65°
Underground tunnels at Sanshandao	−570 m	13186-6		330°	62°
	−570 m	13186-6		330°	50°
	−570 m	13186-6		330°	67°
	−570 m	13186-8		342°	84°
Cangshang open pit	37°21′18.7″	119°54′02.1″	Fig. 2a D1-2	320°	82°
	37°21′19.9″	119°54′02.6″		185°	65°
Underground tunnels at Sanshandao	−570 m	13186-6		315°	75°
	−570 m	13186-8		324°	70°
	−570 m	13186-8		325°	75°
	−570 m	13186-6		180°	75°
Cangshang open pit	37°21′19.9″	119°54′02.6″	Fig. 2b D2-1	70°	68°
Sanshandao surface	37°24′34.9″	119°56′52.1″		66°	62°
	37°24′28.8″	119°56′50.6″		65°	81°
Underground tunnels at Sanshandao	−570 m	13186-6		60°	64°
	−570 m	13186-6		65°	64°
	−570 m	13186-8		65°	61°
	−390 m	1340		66°	62°
	−390 m	1340		75°	73°
	−390 m	1340		80°	77°
Cangshang open pit	37°21′19.9″	119°54′02.6″	Fig. 2 D2-2	110°	80°
Sanshandao surface	37°24′33.9″	119°56′43.6″		105°	76°
	37°24′33.4″	119°56′44.1″		105°	75°
	37°24′33.2″	119°56′47.5″		105°	60°
	37°24′25.0″	119°56′28.1″		110°	75°
	37°24′23.3″	119°56′26.7″		105°	72°
Underground tunnels at Sanshandao	−390 m	1320		105°	76°
	−570 m	13186-6		105°	85°
	−390 m	1320		105°	86°
	−390 m	1320		212°	65°
	−390 m	1340		215°	61°
	−570 m	13186-6		220°	73°
	−390 m	1340		220°	75°
	−390 m	1340		225°	74°

### (U-Th)/He dating

**Figure 3:**
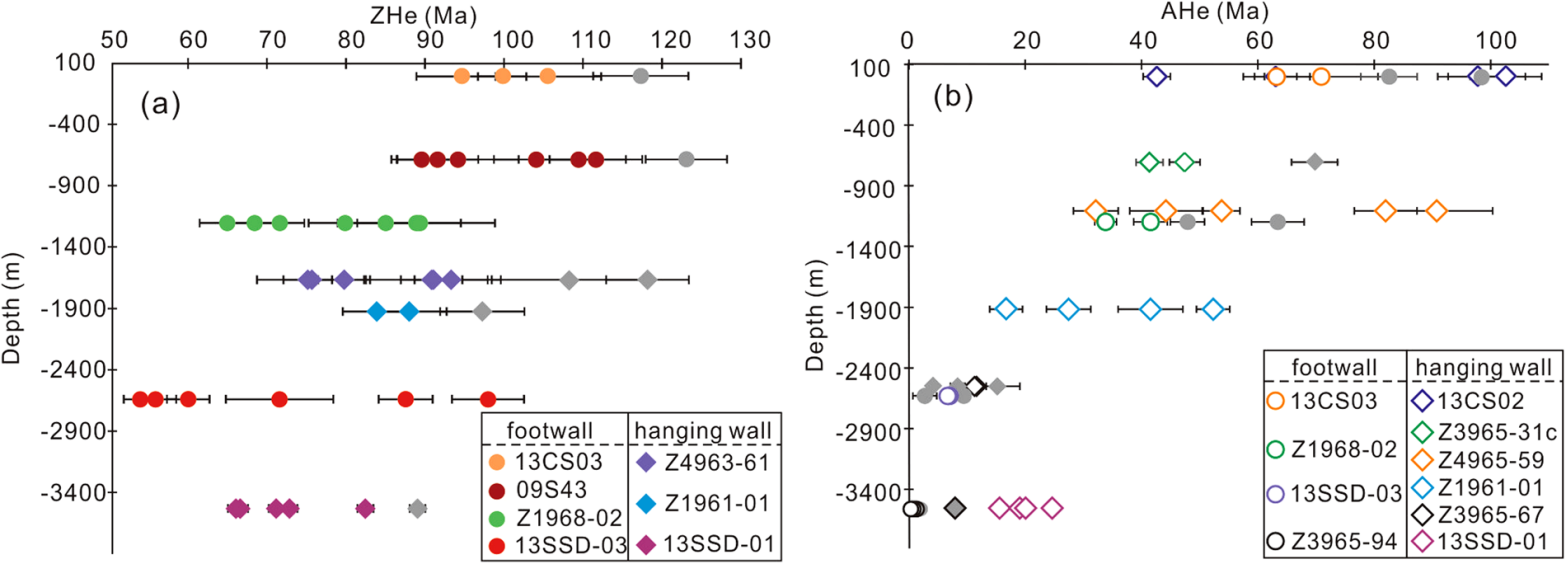
Plots of depths versus ZHe (**d**) and AHe (**e**) ages for the Sanshandao Au deposit. Note that the data in grey are outliers detected by Chauvenet’s criterion.

Overall, 41 single-grain ZHe ages, ranging from 123 Ma to 55 Ma (Fig. 3a; Table 3), were obtained on seven samples (four from footwall and three from hanging wall). Typically, at least three ages were acquired for each sample. In general, these single-grain ages show evident intra-sample variations that exceed analytical precision of <6%. Forty-three single-grain AHe ages were obtained on ten samples (four from footwall and six from hanging wall), ranging from 103 Ma to 0.3 Ma (Fig. 3b; Table 4). These ages also show large intra-sample dispersion, in excess of total analytical uncertainty of 2.5%. In general, the spread becomes less profound with increasing depths. Despite the dispersion, an overall decreasing trend for both ZHe and AHe ages is observed from surface to depth.

**Table 3:** (U-Th)/He analytical results for zircon of the Sanshandao gold deposit.

Sample no.	Depth m	^232^Th ng	Th error %	^238^U ng	U error %	He ncc	He error %	TAU^#^ %	Raw age Ma	± 1σ	Ft	Corrected age Ma	± 1σ	Spherical radius μm	w. ave.** Ma	± 2σ Ma	outlier CVǂ
13CS03 (F)	0	0.69	1.5	1.65	1.9	18.6	1.5	2.3	84.0	1.9	0.72	117.3	6.6	45.11	99.8	5.0	n
		0.17	1.5	1.21	1.9	11.7	1.3	2.3	77.0	1.7	0.81	94.7	6.0	67.83			y
		0.75	1.5	2.14	1.9	23.4	2.0	2.7	82.5	2.2	0.78	105.5	6.7	58.41			y
		0.35	1.5	0.85	2.8	7.6	1.5	2.9	66.8	1.9	0.67	99.9	3.1	39.30			y
09S43 (F)	−680	0.40	1.5	1.18	2.0	10.9	0.8	2.0	69.9	1.4	0.67	104.1	5.4	38.88	98.0	10.0	y
		0.16	1.5	1.07	2.0	10.3	0.8	2.1	75.9	1.6	0.68	111.6	5.9	39.55			y
		0.42	1.5	2.13	2.0	18.0	1.2	2.2	66.1	1.5	0.72	91.6	5.2	45.57			y
		0.29	1.5	1.26	2.0	12.7	0.8	2.0	78.0	1.6	0.71	109.4	6.0	44.23			y
		0.83	1.5	3.18	3.0	26.4	2.6	3.8	64.0	2.4	0.71	89.6	3.8	45.64			y
		0.18	1.6	1.55	2.8	16.6	2.5	3.7	85.0	3.1	0.69	123.2	5.2	42.03			n
		0.22	1.6	1.59	7.7	13.0	2.6	7.9	65.0	5.1	0.69	94.2	7.7	42.06			y
Z1968-02 (F)	−1198	0.53	1.5	4.46	1.9	37.7	2.0	2.8	67.2	1.9	0.76	88.9	10.0	51.89	74.5	8.3	y
		0.44	1.5	8.09	2.0	66.7	0.8	2.1	66.6	1.4	0.75	89.4	5.2	49.50			y
		0.44	1.5	2.17	1.9	16.2	1.6	2.5	58.4	1.4	0.73	79.9	4.6	47.23			y
		0.30	1.5	3.67	2.0	20.6	0.8	2.1	45.1	1.0	0.69	65.0	3.5	41.33			y
		0.41	1.5	9.09	3.0	63.7	2.5	3.8	56.7	2.2	0.79	71.6	3.1	62.68			y
		0.33	1.5	7.98	2.9	67.8	2.5	3.8	68.8	2.6	0.81	85.0	3.6	68.09			y
		0.62	1.5	7.10	3.1	47.0	2.5	3.9	53.1	2.1	0.78	68.5	3.0	58.11			y
Z4963-61 (H)	−1660	0.27	1.5	0.83	1.9	6.5	1.1	2.1	59.8	1.2	0.64	93.3	4.6	35.57	82.5	8.4	y
		0.61	1.5	3.89	1.9	28.9	0.8	2.0	58.5	1.2	0.64	91.1	8.5	35.49			y
		0.29	1.5	0.83	1.9	7.4	1.1	2.1	67.2	1.4	0.62	108.2	9.8	33.65			n
		0.19	1.5	0.32	2.0	2.1	0.8	1.9	46.6	0.9	0.62	75.5	6.8	33.65			y
		0.41	1.5	0.92	2.1	8.4	0.8	2.1	67.7	1.4	0.57	118.2	5.2	30.00			n
		0.08	1.5	2.97	2.8	21.7	2.5	3.8	59.4	2.2	0.65	90.8	3.9	37.63			y
		0.36	1.5	2.41	2.6	14.6	2.5	3.6	47.8	1.7	0.64	75.2	3.1	35.74			y
		1.13	1.5	2.44	2.8	15.7	2.5	3.6	47.6	1.7	0.60	79.8	3.3	32.36			y
Z1961-01 (H)	−1915	0.25	1.5	0.67	2.0	5.0	0.8	1.9	56.0	1.1	0.67	83.9	4.3	38.41	85.7	6.2	y
		0.38	1.5	1.47	1.9	13.3	0.8	1.9	69.7	1.3	0.72	97.2	5.3	44.93			n
		0.25	1.5	1.40	2.0	10.9	0.8	2.0	61.4	1.3	0.70	88.0	4.7	42.02			y
13SSD-03 (F)	−2630	0.14	1.5	0.65	1.9	4.9	1.2	2.1	58.7	1.3	0.60	98.0	4.6	31.70	56.8	7.2	y
		0.09	1.6	0.83	2.0	4.8	1.2	2.3	46.4	1.1	0.65	71.6	6.8	35.99			y
		0.12	1.5	0.53	2.0	2.3	1.2	2.3	33.4	0.8	0.60	55.9	2.6	31.60			y
		0.09	1.6	0.60	1.9	2.6	1.2	2.2	34.4	0.8	0.57	60.0	2.7	29.68			y
		0.14	1.6	0.38	2.9	2.0	2.1	3.4	39.9	1.3	0.74	54.0	2.1	49.88			y
		0.02	4.0	0.56	2.8	3.6	1.8	3.3	51.6	1.7	0.59	87.6	3.4	31.71			y
13SSD-01 (H)	−3520	0.30	1.5	1.64	1.9	10.7	1.2	2.2	51.5	1.1	0.72	71.2	4.0	45.77	73.5	9.3	y
		0.44	1.5	2.21	2.0	15.7	1.2	2.2	55.8	1.2	0.77	72.8	4.4	54.11			y
		0.16	1.5	0.73	1.9	4.5	1.2	2.2	48.2	1.0	0.73	66.1	3.8	46.86			y
		0.16	1.5	1.35	1.9	9.8	1.2	2.2	57.6	1.3	0.65	89.1	4.5	35.85			n
		0.33	1.5	1.68	2.9	12.2	2.6	3.8	57.0	2.2	0.69	82.5	3.5	42.20			y
		0.24	1.5	1.92	3.2	10.9	2.6	4.0	45.3	1.8	0.68	66.6	3.0	40.76			y

Note: F and H after the sample numbers represent footwall and hanging wall.

^*^Represents concentration of the element in femtomole (Th, U in fmol = the analyzed weight in ng/atomic (weight 232 or 238 g/mol) × 10^6^, He in fmol = the analyzed volume in ncc/molar volume for ideal gases (22.4 L/mol) × 10^3^/Ft.

^#^Represent total analytical uncertainty.

^**^Represents weighted average age.

^ǂ^Represents outlier detection by the Chauvenet’s criterion; and “n” indicates outlier and “y” indicates non-outlier.

**Table 4:** (U-Th)/He analytical results for apatite of the Sanshandao gold deposit

Sample no.	Depth m	^232^Th ng	Th error %	^238^U ng	U error %	He ncc	He error %	TAU# %	Raw age Ma	±1σ	Ft	Corrected age Ma	±1σ	Spherical radius μm	outlier CVǂ
13CS02 (H)	0	0.085	8.35	0.069	6.68	0.90	2.12	5.90	82.05	4.84	0.80	102.6	6.1	76.93	y
		0.039	5.50	0.060	4.61	0.60	2.60	4.82	70.95	3.42	0.73	97.8	5.1	55.36	y
		0.037	5.50	0.035	4.99	0.26	3.49	5.42	47.91	2.60	0.76	63.1	3.6	63.90	y
		0.060	5.44	0.020	4.58	0.14	3.70	5.10	32.30	1.65	0.76	42.6	2.3	65.25	y
13CS03 (F)	0	0.018	7.96	0.007	4.74	0.06	7.15	8.30	42.12	3.49	0.43	98.4	9.1	27.51	n
		0.006	6.48	0.008	4.65	0.04	10.8	11.5	33.48	3.86	0.53	63.2	7.5	32.48	y
		0.006	6.49	0.006	4.86	0.03	12.5	13.2	34.03	4.49	0.48	70.9	9.8	29.42	y
		0.009	7.88	0.011	4.61	0.09	4.71	6.24	52.37	3.27	0.63	82.6	5.7	41.65	n
Z3965-31c (H)	−706	0.079	5.43	0.040	4.86	0.25	3.66	5.23	34.14	1.79	0.72	47.4	2.7	55.68	y
		0.049	5.45	0.013	4.61	0.16	3.13	4.73	54.63	2.58	0.78	69.8	3.6	73.33	n
		0.075	5.45	0.023	4.65	0.16	3.07	4.68	32.44	1.52	0.78	41.3	2.1	73.54	y
Z4965-59 (H)	−1107	0.031	5.87	0.014	4.94	0.10	3.33	5.06	36.47	1.85	0.68	53.8	3.2	48.72	y
		0.018	5.58	0.007	4.57	0.03	11.1	11.7	19.83	2.31	0.62	32.1	3.9	41.03	y
		0.050	5.57	0.007	4.67	0.09	4.35	5.85	40.33	2.36	0.49	81.9	5.4	31.95	y
		0.003	13.7	0.008	4.68	0.02	13.1	13.8	22.87	3.15	0.52	44.2	6.2	31.21	y
		0.000	37.5	0.006	4.58	0.03	9.10	10.2	47.37	4.82	0.52	90.7	9.6	31.21	y
Z1968-02 (F)	−1198	0.009	5.78	0.032	5.05	0.14	5.01	6.91	34.18	2.36	0.82	41.6	2.9	84.56	y
		0.051	5.47	0.137	5.05	0.50	2.81	5.44	27.67	1.51	0.82	33.8	1.9	82.73	y
		0.007	6.35	0.053	4.64	0.33	3.06	5.45	48.87	2.66	0.77	63.4	3.7	65.23	n
		0.003	8.64	0.054	4.89	0.25	3.15	5.76	37.69	2.17	0.79	47.9	2.9	69.78	n
Z1961-01 (H)	−1915	0.015	5.90	0.021	4.95	0.11	2.66	5.08	37.33	1.90	0.71	52.3	2.9	53.11	y
		0.013	6.55	0.011	4.57	0.03	12.9	13.5	17.47	2.35	0.64	27.5	3.8	42.21	y
		0.003	10.6	0.010	5.08	0.03	12.1	13.0	24.85	3.24	0.60	41.5	5.6	37.43	y
		0.006	12.9	0.018	4.72	0.03	16.0	16.6	10.55	1.75	0.63	16.7	2.8	40.70	y
Z3965-67 (H)	−2551	0.006	6.60	0.012	4.63	0.01	16.7	17.2	7.38	1.27	0.65	11.3	2.0	43.38	y
		0.002	14.9	0.006	4.67	0.002	7.07	8.36	2.59	0.22	0.62	4.2	0.4	39.93	n
		0.018	5.62	0.018	4.65	0.01	14.3	14.8	5.21	0.77	0.62	8.4	1.3	40.32	n
		0.005	8.11	0.006	4.75	0.01	2.36	4.84	6.54	0.32	0.56	11.7	0.7	34.70	y
		0.003	11.7	0.007	4.61	0.01	25.0	25.4	8.64	2.19	0.57	15.2	3.9	34.89	n
13SSD-03 (F)	−2630	0.043	5.46	0.013	4.60	0.01	15.4	15.8	4.59	0.73	0.69	6.7	1.1	50.72	y
		0.130	5.43	0.011	4.63	0.01	22.3	22.6	1.78	0.40	0.65	2.7	0.6	46.46	n
		0.012	5.69	0.011	4.65	0.01	28.6	28.9	4.14	1.20	0.59	7.0	2.0	37.37	y
		0.012	6.25	0.011	4.61	0.01	20.0	20.4	5.79	1.18	0.61	9.5	1.9	39.52	n
13SSD-01 (H)	−3520	0.019	6.05	0.041	4.63	0.08	5.13	6.65	14.02	0.93	0.74	19.1	1.3	57.15	y
		0.038	5.56	0.061	4.72	0.13	3.20	5.26	14.63	0.77	0.73	20.0	1.1	56.16	y
		0.002	23.5	0.017	4.66	0.01	18.2	18.8	5.30	0.99	0.67	7.9	1.5	44.91	n
		0.036	5.45	0.087	4.64	0.15	3.66	5.62	12.63	0.71	0.81	15.6	0.9	80.09	y
		0.144	5.42	0.065	4.56	0.24	3.86	5.23	19.90	1.04	0.81	24.6	1.3	81.77	y
Z39965-94 (F)	−3563	0.057	5.50	0.024	4.57	0.001	14.1	14.6	0.22	0.03	0.57	0.4	0.1	36.41	y
		0.042	5.58	0.012	4.64	0.003	4.71	5.92	1.13	0.07	0.59	1.9	0.1	38.65	n
		0.011	6.04	0.013	4.60	0.001	14.1	14.7	0.52	0.08	0.65	0.8	0.1	43.25	y
		0.051	5.44	0.018	4.59	0.003	4.71	5.88	0.83	0.05	0.63	1.3	0.1	42.24	y
		0.072	5.44	0.022	4.69	0.001	14.1	14.6	0.21	0.03	0.65	0.3	0.0	45.84	y

*Represents concentration of the element in femtomole (Th, U in fmol = the analyzed weight in ng/atomic weight (232 or 238 g/mol) × 10^6^, He in fmol = the analyzed volume in ncc/molar volume for ideal gases (22.4 L/mol) × 10^3^/Ft.

^**^Represents weighted average age.

^ǂ^Represents outlier detection by the Chauvenet’s criterion; and “n” indicates outlier and “y” indicates non-outlier.

### Outlier detection

Dispersion of (U-Th)/He ages is not uncommon in natural samples. To avoid the potential influence of “outliers” on interpretation, an evaluation procedure applying Chauvenet’s criterion were conducted. This method has been applied as a tool for outlier detection in (U-Th)/He thermochronology previously^22^, the idea being that all data points from a normally distributed population should lie within a probability of 1-1/(2n) (n is the sample size) centering on the mean, and that data point falling outside this range can be discarded. The assumption of normality of our data was assessed by Quantile-Quantile plot^23^. The correlation coefficients for the plots are larger than 0.93, indicating the data follow a normal distribution pattern, which validates the use of Chauvenet’s criterion.

## Discussion

### Causes of outliers and age dispersion

**Figure 4:**
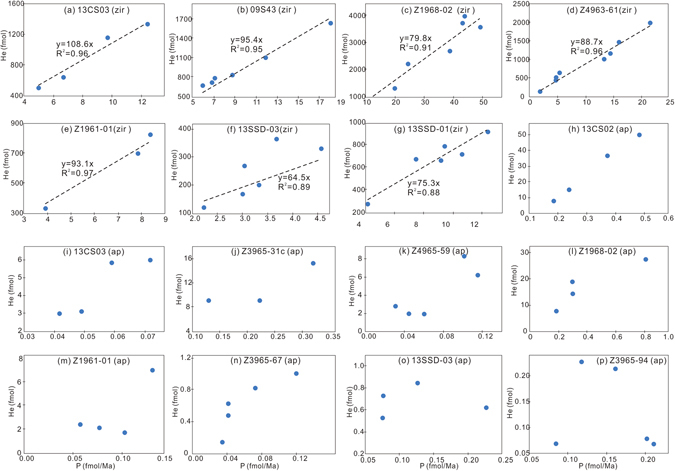
Plots of helium concentration versus production rate (P) for zircon and apatite from Sanshandao. zir: zircon; ap: apatite.

Six ZHe ages failed the Chauvenet’s criterion test (Fig. 2d,e). These outliers are the oldest dates within the single dataset, and several (e.g. 123 Ma of the sample 09S43) are close to the mineralization age (120 Ma). Normally, when an abnormally old He age is obtained, the presence of “excess” non-radiogenic helium daughters is suspected. Sources of excess He include insoluble U-Th-rich mineral (more likely in AHe dating where simple acid dissolution is utilized), He-trapping inclusions^24^ or, in rare circumstances, implantation of He by a neighboring U-Th rich mineral^25^. If these occur, a poorly defined isochron^26^ is obtained on a plot of corrected He content versus production rate. However, all samples but 13SSD03 show good, well-defined linear correlations (Fig. 4a–g) so the cause of the older ZHe ages remains unclear. Another notable observation is that all older ZHe age outliers came from Linglong granite zircons, but no “too old” ages were obtained from zircon in the Guojialing granodiorite despite the fact that all samples were hydrothermally altered by mineralizing fluids with temperatures of up to 400 °C^27^. For the purpose of investigating the post-mineralization thermal history, the ZHe outliers are excluded from further interpretation.

For the remaining data, the ZHe ages overlap within 1σ error except for 13SSD-03, which may have been affected by excess He. For clustered ages, a weighted mean value was calculated by *Isoplot* 4 to represent the true age of the sample while the youngest age of 54 Ma is regarded as the best estimate for 13SSD-03.

**Figure 5:**
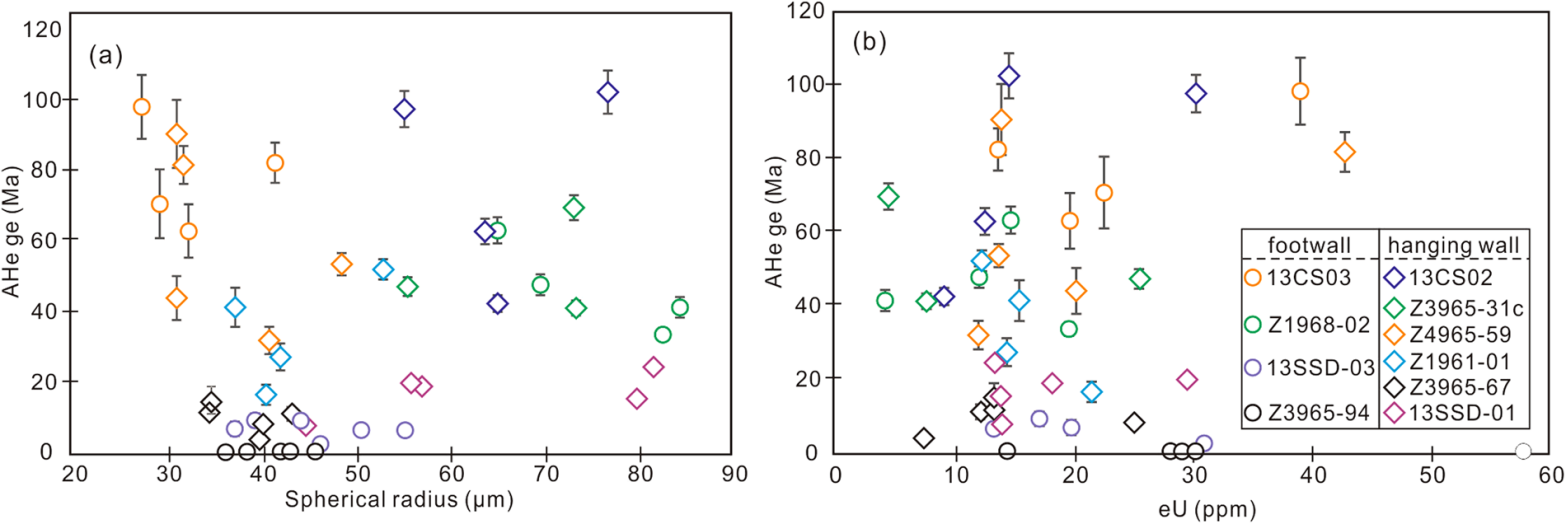
(**a**) A plot of AHe ages versus spherical radius for each grain; (**b**) A plot of AHe ages versus eU concentration (eU = U + 0.235Th). Note that for each sample AHe ages are neither positively related to grain sizes nor eU concentrations. Symbols in (**a**) are the same as in (**b**).

Twelve AHe ages were identified as outliers using Chauvenet’s criterion. Unlike the ZHe ages, the outliers are from both Linglong and Guojialing samples. Plausible explanations for the age dispersion include crystal fragmentation^28^, heterogeneous distribution of parental nuclides^29, 30^ that complicates routine α-ejection correction, presence of undetected U-Th or He-bearing inclusions^31^, differences in grain size^32^, implantation of He from neighboring U-Th rich phases^33^ and radiation damage that changes He retentivity and closure temperature^34^. In this study, the effect of fragmentation is excluded since all analyzed mineral grains were complete crystals. Difference in grain size and radiation damage is not considered responsible because no positive relationship between AHe ages and grain size and eU values was observed (Fig. 5a,b). The possibility of He implantation is ruled out based on the analysis of petrographic thin sections in which no U-Th rich phases were observed near apatite crystals. It is, however, impossible to evaluate the influence of parental nuclide heterogeneity due to a lack of related data. The laser ablation ICPMS study by Farley *et al*.^35^ demonstrated that a 15% age difference can be introduced between rim U-enriched and core U-enriched apatites. Even if Sanshandao apatites have parental distribution patterns as extreme as the grain Farley *et al*. analyzed, the expected age variations are unable to fully account for the dispersion observed in Sanshandao AHe ages (>50%).

Having ruled out the above possibilities, the presence of inclusions seems the most likely explanation. Most of the apatite grains obtained from the samples were milky, making detection of micro-inclusions impossible, and where the grains were clear enough, black or transparent inclusions were often observed. While every effort was taken to avoid analysis of grains with mineral inclusions, it is likely that some inclusions were undetected during pre-analysis optical microscopic inspection. The influence of inclusion on AHe ages is clearly shown by the scatter on the plots of corrected He content versus production rate (Fig. 4h–p). For 13SSD-01, the apatite grains analyzed contain abnormally high He contents for a sample currently sitting at a depth of 3.5 km (corresponding to a temperature of nearly 100 °C), and, therefore, this sample was not used for interpretation of results. The youngest ages are used in the following interpretation.

### Thermal history and exhumation magnitude

**Figure 6:**
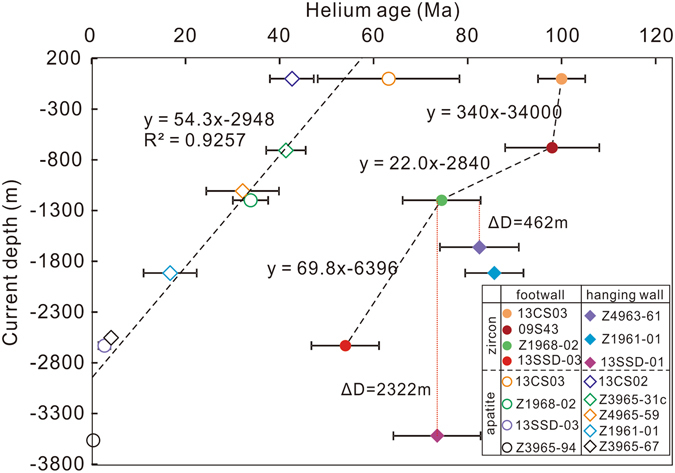
A plot of ZHe and AHe ages versus depth for rock samples from the footwall and hang wall of the Sanshandao Au deposit. The ZHe ages are weighted mean ages and 2σ errors from Table 3 after discarding the outliers, and the youngest value of 54 Ma was used due to influence of inclusions. The AHe ages are the youngest single age from each sample and the errors are 2σ. Note ZHe ages from hanging wall are older than those from similar depth of footwall; while AHe ages are indistinguishable for hanging wall and footwall, and form a good linear array. See the text for detailed interpretation.

One of the main purposes of this study is to establish the thermal history after the formation of ore bodies in the Sanshandao Au deposit. Although the data acquired are quite scattered and complex, the data cleansing procedure makes it possible to extract valid and versatile information from the ZHe and AHe data as shown in Fig. 6. The ZHe age profile exhibits two broad features; (1) the (U-Th)/He ages decrease with depth (as shown by the slope of the regression lines in Fig. 6); (2) ZHe ages from hanging wall samples are older than ZHe ages from similar depths in the footwall. The second feature is associated with fault offset and will be discussed in detail in the next section.

Between the surface and −680 m, the ZHe ages are invariant, which likely indicates a phase of rapid cooling through the He partial retention zone of zircon (ZHePRZ) in the late Early Cretaceous. This cooling phase could be related to the cooling of the ore-forming fluids to the ambient temperature of wall rocks. Alternatively, a rapid exhumation along a normal fault at ca. 110 Ma might be a more viable explanation^36^. Between −680 m −1200 m, the ZHe ages decrease from 100 Ma to 75 Ma. The moderate decrease is explained by a slow cooling or a thermal quiescence in the early Late Cretaceous (100‒75 Ma), when the samples resided in the HePRZ and may have acquired a wide range of ZHe ages. The origin of this thermal episode might be attributed to the NW-SE compression at around 100 Ma. This is consistent with the tectonic model proposed by Zhang *et al*.^37^, which suggests a major uplift yet minor amount of erosion or exhumation in the East Asia coastal mountains. The magnitude of the last age variation between −1200 m and −2660 m is intermediate in magnitude between the first and second cooling phases, and this change likely reflects an episode of revived cooling between 75 Ma and 55 Ma. The late phase of normal faulting might be partly responsible for this period of cooling. Additionally, the Jiaolai Basin, immediately south of the deposit, received up to 10 km thickness of sediments sourced from the Jiaobei Uplift as evidenced by a detrital zircon study^38^. The intense sedimentation of the basin argues strongly for an intense erosion process within the northern terrane. On a larger scale, this period of time also witnessed a major crustal subsidence of the coastal mountain-basin system in eastern Asia^37^. Therefore, the combined effect of the topographic contrast between the Jiaobei Uplift and Jiaolai Basin, as well as between the coastal mountain and surrounding regions could have been the primary cause for the rapid cooling recorded by ZHe ages.

It is impossible to extract any information about post-Eocene thermal history from ZHe ages due to a lack of samples from deeper levels, and this piece of information is supplemented by the AHe ages that are sensitive to much lower temperatures. The first striking feature about the AHe dataset is that the oldest AHe ages (45–60 Ma; samples 13CS02 and 13CS03) overlap with the youngest ZHe age (54 Ma; sample 13SSD-03), and the current vertical distance between these samples is about 3 km. This observation is consistent with the observation that the ZHePRZ lies about 3–5 km deeper than the HePRZ of apatite (AHePRZ)^6, 36^. In addition, this feature suggests that our AHe dataset records the latest thermal episodes, beyond the temperature range of the ZHe thermochronometer. A closer examination of the distribution profile (Fig. 6) revealed another two features: (1) there is an absence of discernable difference between the AHe ages of samples from the footwall and hanging wall, the significance of which will be discussed in the following section; and (2) most AHe ages fall along a linear array that argues strongly for the existence of a monotonic cooling^7^ since the Eocene and suggests an exhumation rate of 54.3 m/Myr. This period of slow cooling and the similarity of AHe ages between the fault walls reflects a lack of activity along the SCF and tectonic quiescence in the Jiaodong region^38^, although Wu *et al*.^39^ suggested a major exhumation event at around 45 Ma for the Sulu belt and eastern Jiaodong province.

In summary, post to the gold mineralization at ca. 120 Ma, the Sanshandao Au deposit might have experienced an early cooling at ca. 100 Ma corresponding to normal faulting along the SCF, which was followed by a short thermal quiescence for the next 25 Myr. The cooling process revived in response to a combination of fault offset and regional erosion between 75 Ma and 55 Ma. Since Eocene, the deposit underwent quite constant cooling in association with a protracted erosion process. It is evident that at least the last two phases of cooling are linked to tectonic denudation or erosion.

An estimation of Eocene exhumation is quite straightforward because of the well-defined exhumation rate from 40‒60 Ma to 0 Ma (54.3 m/Myr). Assuming a length of ﻿55 Myr of exhumation, which likely marks the end of the Late Cretaceous cooling, 3.0 km of exhumation may have occurred. A similar calculation for Late Cretaceous cooling (75 Ma to 55 Ma) is not as straightforward because it is not possible to define a clear age pattern with only two ZHe ages. However, the slope of this section in Fig. 6 (69.8 m/Myr) may represent a minimum estimate of the exhumation rate based on the observation that structural and erosional processes were intense^14^. Thus, over a period of 20 Myr (75 Ma to 55 Ma), 1.4 km of exhumation may have occurred. In order to roughly estimate the exhumation magnitude associated with Early Cretaceous cooling (around 100 Ma) given limited data, we can take the distance between surface sample 13CS03 and sample 09S43 (680 m) to provide a minimum estimate of the exhumation. Overall, we conclude that the Sanshandao gold deposit may have exhumed at least 5.1 km.

### Timing and magnitude of fault displacement

This study also investigated the timing and magnitude of fault displacement along the SCF after Au mineralization. Structural documentation and paleostress reconstruction revealed two phases of normal movement and one intervening phase of sinistral movement. A comparison with the temporal evolution of the regional stress field and kinetic history of the adjacent JXF enables us to infer the timing for the two phases of normal displacement. Theoretically, if these structural events occurred at temperatures close to, or below the thermal window at which (U-Th)/He thermometers are sensitive, older ages would be observed in hanging wall samples relative to those at the same depth in the footwall because samples at higher levels accumulated He longer^5^. As mentioned earlier, our ZHe ages from hanging wall samples are older than ZHe ages from similar depths in the footwall. This excursion explicitly demonstrates the presence of fault displacement along the SCF. Interestingly, the fault displacement was not recorded by the AHe ages, likely indicating that displacement occurred before the apatite crystals were cooled below the closure temperature (i.e. ca. 40–60 °C), confirming the interpretation from structural considerations. It is difficult to obtain an unambiguous estimate of displacement magnitude without the ability to clearly define the age patterns for both fault walls, however, a rough estimation can be made by fitting identical ages between the fault walls and calculating the depth difference. Taking footwall sample Z1968 (−1198 m) as the reference value, if the age of hanging wall sample Z4963–61 (−1660 m) is considered identical, then a depth difference of 462 m is obtained (Fig. 6). Similarly, sample 13SSD-01 (−3520 m) is 2322 m deeper than Z1968 (Fig. 6). We tentatively suggest that the fault may have been vertically offset between 0.5 km and 2.3 km.

### Exploration implications

Our calculations indicate that Sanshandao has been exhumed ca. 5.1 km since its formation. This estimation is in consistent with previous calculations based on fluid inclusion thermobarometry which indicated that the deposit was formed at a depth of >4 km^8^. Groves *et al*.^40^ suggested that ore bodies in orogenic fault-controlled gold deposits may extend to depths of several kilometers. An important implication of this estimation is that part of the original orebodies has been removed by erosion, and that large reserves could still exist at depth. In fact, this inference is consistent with the recent exploration drilling project which revealed economic orebodies down to −2600 m. Equally important, if a similar exhumation magnitude and formation depth fits the entire Jiaobei Uplift, shallowly-emplaced ore deposits such as epithermal and porphyry ore deposits that formed pre-Cretaceous might have been largely eroded. This is consistent with the observation that very few epizonal deposits have been discovered in the Jiaodong province.

## Methods

(U-Th)/He dating on zircon and apatite was completed in the John de Laeter Center, Curtin University. Preliminary grain selection targeted at those euhedral and complete zircon and apatite crystals with a binocular microscope, and then individual grains were carefully examined under plain and cross-polarized light for mineral or fluid inclusions that may contribute excess helium. Measurements of the long and short axis of each grain were used for later calculation of the alpha correction factor (Ft)^41^. Characterized zircon and apatite grains were loaded into niobium and platinum microvials, respectively, for helium extraction. Helium was thermally extracted from each individual encapsulated crystal using a diode laser. ^4^He abundances were measured by isotope dilution method using a pure ^3^He spike, calibrated daily against an independent ^4^He standard tank.

After degassing, the U and Th contents of the zircon grain were determined using an isotope dilution ICP-MS. Samples were removed from the laser chamber and transferred to Parr pressure dissolution vessels where they were spiked with ^235^U and ^230^Th (25 μl of a solution containing 15 ppb ^235^U and 15 ppb ^230^Th) and digested at 240 °C for 40 hours in 350 μl of HF. Standard solutions were spiked and treated similarly, as were a series of unspiked reagent blanks. After digestion, the solutions were removed from the pressure vessels and dried for 2 days. HCl acids (300 μl) was added to each vial, which was then subjected to a second bombing for 24 hours at 200 °C to ensure dissolution of fluoride salts. Final solutions were diluted to 10% acidity for analysis on an Agilent 7500CS mass spectrometer (TSW™ Analytical). For single crystals digested in small volumes (0.3–0.5 ml), U and Th isotope ratios were measured at a precision of <2%^41^. Repeated measurement of internal age zircon standards by (U-Th)/He methods at Curtin has an estimated precision of <6%.

For degassed apatite grains, their U and Th contents were determined by isotope dilution using ^235^U and ^230^Th spikes. 25 μl of a 50% (by volume; approximately 7 M) HNO_3_ solution containing approximately 15 ppb ^235^U and 5 ppb ^230^Th was added to each sample. The apatite was digested in the spiked acid for at least 12 hours to allow equilibrium between the spike and sample isotopes. Standard solutions containing 27.6 ppb U and 28.4 ppb Th, were spiked and treated identically to samples, as were a series of unspiked reagent blanks. 250 μl of Milli-Q water was added prior to analysis on an Agilent 7500CS mass spectrometer. U and Th isotope ratios were determined at a precision of <2%. Overall apatite (U-Th)/He thermochronology analysis at Curtin has a precision of 2.5%, based on multiple age determinations (n = 26) of Durango standard which produce an average age of 31.1 ± 1.0 (2σ) Ma.

## References

[1] Zeitler PK, Herczeg AL, McDougall I, Honda M (1987). U-Th-He dating of apatite: A potential thermochronometer. Geochim Cosmochim Ac.

[2] Danišík M, Štěpančíková P, Evans N (2012). Constraining long-term denudation and faulting history in intraplate regions by multisystem thermochronology: An example of the Sudetic Marginal Fault (Bohemian Massif, central Europe). Tectonic..

[3] Crowhurst PV, Green PF, Kamp PJJ (2002). Appraisal of (U-Th)/He apatite thermochronology as a thermal history tool for hydrocarbon exploration: An example from the Taranaki Basin, New Zealand. AAPG Bull.

[4] McInnes BIA, Farley KA, Sillitoe RH, Kohn BP (1999). Application of apatite (U-Th)/He thermochronometry to the determination of the sense and amount of vertical fault displacement at the Chuquicamata porphyry copper deposit, Chile. Econ Geol.

[5] McInnes BIA, Evans NJ, Fu FQ, Garwin S (2005). Application of thermochronology to hydrothermal ore deposits. Rev Mineral Geochem.

[6] Reiners PW (2005). Zircon (U-Th)/He Thermochronometry. Rev Mineral Geochem.

[7] Wolf RA, Farley KA, Kass DM (1998). Modeling of the temperature sensitivity of the apatite (U-Th)/He thermochronometer. Chem Geol.

[8] Fan HR, Zhai MG, Xie YH, Yang JH (2003). Ore-forming fluids associated with granite-hosted gold mineralization at the Sanshandao deposit, Jiaodong gold province, China. Miner Deposita.

[9] Goldfarb RJ, Santosh M (2014). The dilemma of the Jiaodong gold deposits: are they unique?. Geosci Front.

[10] Zheng JP, Sun M, Lu FX, Pearson N (2003). Mesozoic lower crustal xenoliths and their significance in lithospheric evolution beneath the Sino–Korean Craton. Tectonophysics.

[11] Xie S (2012). U-Pb ages and trace elements of detrital zircons from Early Cretaceous sedimentary rocks in the Jiaolai Basin, north margin of the Sulu UHP terrane: provenances and tectonic implications. Lithos.

[12] Zhou T, Lyu G (2000). Tectonics, granitoids and Mesozoic gold deposits in East Shandong, China. Ore Geol Rev.

[13] Yang JH, Wu FY, Wilde SA (2003). A review of the geodynamic setting of large-scale Late Mesozoic gold mineralization in the North China Craton: an association with lithospheric thinning. Ore Geol Rev.

[14] Zhang YQ, Li JL, Zhang T, Dong SW, Yuan JY (2008). Cretaceous to Paleocene tectono-seidimentary evolution of the Jiaolai Basin and the contiguous areas of the Shandong peninsula (North China) and its geodynamic implications. Ac Geol Sinica.

[15] Deng J, Wang C, Bagas L, Carranza EJM, Lu Y (2015). Cretaceous–Cenozoic tectonic history of the Jiaojia Fault and gold mineralization in the Jiaodong Peninsula, China: constraints from zircon U-Pb, illite K-Ar, and apatite fission track thermochronometry. Miner Deposita.

[16] Song MC (2015). Discovery and tectonic-magmatic background of superlarge gold deposit in offshore of northern Sanshandao, Shandong peninsula, China. Ac Geol Sinica.

[17] Li XC (2013). Hydrothermal alteration associated with Mesozoic granite-hosted gold mineralization at the Sanshandao deposit, Jiaodong Gold Province, China. Ore Geol Rev.

[18] Yang KF (2012). Reactivation of the Archean lower crust: implications for zircon geochronology, elemental and Sr–Nd–Hf isotopic geochemistry of late Mesozoic granitoids from northwestern Jiaodong Terrane, the North China Craton. Lithos.

[19] Dou JZ, Fu S, Zhang HF (2015). Consolidation and cooling paths of the Guojialing granodiorites in Jiaodong Peninsula: Implication for crustal uplift and exhumation. Ac Petrol Sinica.

[20] Zhang XO (2003). Geology and timing of mineralization at the Cangshang gold deposit, north-western Jiaodong Peninsula, China. Miner Deposita.

[21] Hu FF (2013). Fluid inclusions at different depths in the Sanshandao gold deposit, Jiaodong Peninsula, China. Geofluids.

[22] Fitzgerald PG, Baldwin SL, Webb LE, O’Sullivan PB (2006). Interpretation of (U-Th)/He single grain ages from slowly cooled crustal terranes: a case study from the Transantarctic Mountains of southern Victoria Land. Chem Geol.

[23] Wilk MB, Gnanadesikan R (1968). Probability plotting methods for the analysis of data. Biometrika.

[24] Danišík M (2017). Seeing is believing: Visualization of He distribution in zircon and implications for thermal history reconstruction on single crystals. Sci Adv.

[25] Spencer, A. S. *et al*. The importance of residing in a good neighbourhood: rechecking the rules of the game for apatite (U-Th)/He thermochronology. In: Andressien, P. (Ed.), 10th International Fission track Dating Conference. Amsterdam, pp. 20 (2004).

[26] Vermeesch P (2008). Three new ways to calculate average (U-Th)/He ages. Chem Geol.

[27] Wen BJ (2016). Fluid evolution and ore genesis of the giant Sanshandao gold deposit, Jiaodong gold province, China: Constrains from geology, fluid inclusions and H–O–S–He–Ar isotopic compositions. J Geochem Explor.

[28] Brown RW (2013). Natural age dispersion arising from the analysis of broken crystals. Part I: Theoretical basis and implications for the apatite (U-Th)/He thermochronometer. Geochim Cosmochim Acta.

[29] Meesters AGCA, Dunai TJ (2002). Solving the production–diffusion equation for finite diffusion domains of variousshapes: Part II. Application to cases with a-ejection and nonhomogeneous distribution of the source. Chem Geol.

[30] Hourigan JK, Reiners PW, Brandon MT (2005). U-Th zonation dependent alpha-ejection in (U-Th)/He chronometry. Geochim Cosmochim Ac.

[31] House MA, Wernicke BP, Farley KA, Dumitru TA (1997). Cenozoic thermal evolution of the central Sierra Nevada, California, from (U Th)/He thermochronometry. Earth Planet Sc Lett.

[32] Farley KA (2000). Helium diffusion from apatite: General behavior as illustrated by Durango fluorapatite. J Geophys Res.

[33] Danišík M (2010). Tectonothermal history of the Schwarzwald Ore District (Germany): An apatite triple dating approach. Chem Geol.

[34] Shuster DL, Farley KA (2009). The influence of artificial radiation damage and thermal annealing on helium diffusion kinetics in apatite. Geochim Cosmochim Ac.

[35] Farley KA, Shuster DL, Ketcham RA (2011). U and Th zonation in apatite observed by laser ablation ICPMS, and implications for the (U-Th)/He system. Geochim Cosmochim Acta.

[36] Stockli DF (2005). Application of low-temperature thermochronometry to extensional tectonic settings. Rev Mineral Geochem.

[37] Zhang L (2016). High elevation of Jiaolai Basin during the Late Cretaceous: Implication for the coastal mountains along the East Asian margin. Earth Planet Sc Lett.

[38] Zhou JB, Han W, Song MC (2016). The exhumation of the Sulu Terrane and the forming of the Tancheng-Lujiang Fault: evidence from detrital zircon U-Pb dating of the Mesozoic sediments of the Laiyang basin, Central China. Ac Petrol Sinica.

[39] Wu L (2016). Cenozoic exhumation history of Sulu terrane: Implications from (U-Th)/He thermochronology. Tectonophysics.

[40] Groves DI, Goldfarb RJ, Gebre-Mariam M, Hagemann SG, Robert F (1998). Orogenic gold deposits: a proposed classification in the context of their crustal distribution and relationship to other gold deposit types. Ore Geol Rev.

[41] Evans NJ, Wilson NSF, Cline JS, McInnes BIA, Byrne J (2005). Fluorite (U-Th)/He thermochronology: Constraints on the low temperature history of Yucca Mountain, Nevada. Appl Geochem.

